# Viral Etiology of Acute Respiratory Infection in Gansu Province, China, 2011

**DOI:** 10.1371/journal.pone.0064254

**Published:** 2013-05-14

**Authors:** Guohong Huang, Deshan Yu, Naiying Mao, Zhen Zhu, Hui Zhang, Zhongyi Jiang, Hongyu Li, Yan Zhang, Jing Shi, Shuang Zhang, Xinhua Wang, Wenbo Xu

**Affiliations:** 1 Key Laboratory of Medical Virology Ministry of Health, National Institute for Viral Disease Control and Prevention, Chinese Center for Disease Control and Prevention, Beijing, People's Republic of China; 2 Gansu Center for Disease Control and Prevention, Lanzhou, People's Republic of China; 3 Xinjiang Medical University, Urumqi, People's Republic of China; University of Hong Kong, Hong Kong

## Abstract

**Background:**

Acute respiratory infections (ARIs) are the leading cause of children and their leading killer. ARIs are responsible for at least six percent of the world's disability and death. Viruses are one of the most common agents causing ARIs. Few studies on the viral etiology and clinical characteristics of ARIs have been performed in the northwest region of China, including Gansu Province.

**Methods:**

Clinical and demographic information and throat swabs were collected from 279 patients from January 1st to December 30st, 2011. Multiplex RT-PCR was performed to detect 16 respiratory viral pathogens.

**Results:**

279 patients were admitted for ARIs. The patients aged from 1 month to 12 years, with the median age of 2 years. Of which, 105 (37.6%) were positive for at least one pathogen. A total of 136 respiratory viral pathogens were identified from the 105 patients. Respiratory syncytial virus (RSV) was the most frequently detected pathogen (26.5%, 36/136), followed by parainfluenza virus (PIV) 1–3 (22.1%, 30/136), human rhinovirus (HRV) (21.3%, 29/136), human coronavirus (CoV) (10.3%, 14/136) and human adenovirus (HAdV) (9.6%, 13/136). Influenza A (Flu A), human metapneumovirus (hMPV) and human bocavirus (BoCA) were found 4.4%, 3.7% and 2.2%, respectively. Influenza B (Flu B) and seasonal influenza A H1N1(sH1N1) were not detected. Single-infections were detected in 30.5% (85/279) of cases. RSV was the most common pathogens in patients under 1 year and showed seasonal variation with peaks during winter and spring.

**Conclusions:**

This paper presents data on the epidemiology of viral pathogens associated with ARIs among children in Gansu Province, China. RSV is most frequently detected in our study. The findings could serve as a reference for local CDC in drawing up further plans to prevent and control ARIs.

## Introduction

Acute respiratory infections (ARIs) are a group of diseases that include pneumonia, influenza, and respiratory syncytial virus (RSV). According to data from the World Health Organization (WHO), ARIs are responsible for at least six percent of the world's disability and death and cause a world disease burden of 94,037,000 (in terms of disability-adjusted life years (DALYs)) [1]. In terms of geographical distribution, 70% of children who died from ARIs are in Africa and Southeast Asia [2]. Respiratory syncytial virus (RSV), influenza A and B viruses (Flu A and B), human coronavirus (CoV), human rhinoviruses (HRV), human adenoviruses (HAdV), human metapneumovirus (hMPV), and human bocavirus (BoCA) have been identified as the common causes of ARIs among populations [3], [4].

Although the incidence of ARIs could be similar around the world, and several groups have reported the prevalence and clinical presentation of viral infections in China, studies conducted in remote and less well developed areas of China are scarce. This paper presents data on the epidemiology of viral pathogens associated with ARI among children in Gansu Province, which located in a relatively undeveloped area of China. It aims to provide basic data to direct local disease prevention and control measures.

## Results

### Patient characteristics

From January 1st to December 31st, 2011, 279 eligible patients with ARIs were enrolled in this study. Ages ranged from 1 month to 12 years (median  = 2 years) and 153(54.8%, N = 279) patients were aged between 1 and 6 years old. 183(65.6%, N = 279) were male (M/F ratio  = 1.9). The distribution of respiratory viruses in males and females had no difference (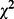 = 0.009, P = 0.92). Demographic and clinical characteristics of patients are shown in Table 1. Among the 279 patients, 94.6% (264/279) suffered from fever, which was the most common clinical symptom, followed by cough (89.3%, 249/279) and expectoration (49.8%, 139/279). 39.4% (110/279) of patients showed abnormal chest radiography. The average hospital stay duration was 6 days.

**Table 1:** Demographic and clinical characteristics of patients presenting ARIs.

Characteristics	Total (%) N = 279	Virus-positive (%) N = 105
**Sex**		
Male	183(65.6)	69(65.7)
Female	96(34.4)	36(34.3)
**Age groups(years)**		
<1	88(31.5)	29(27.6)
1–6	144(51.6)	63(60.0)
>6	47(16.8)	13(12.4)
**Clinical symptoms**		
Fever	264(94.6)	101(96.2)
Sore throat	52(18.6)	24(22.9)
Cough	249(89.3)	96(91.4)
Dyspnea	19(6.8)	5(4.8)
Polypnea	23(8.2)	8(7.6)
Chest pain	8(2.9)	0(0)
Expectoration	139(49.8)	50(47.6)
Moist rales	32(47.3)	47(44.8)
Dry Rales	20(7.2)	8(7.2)
Abnormal chest radiography	110(39.4)	41(39.0)
Hospital duration	6(5–9)	6(5–8)

### Viral etiologies

105 (37.6%, N = 279) patients were positive for at least one pathogen. Single-infections accounted for 30.5% (85/279) of cases, co-infections were found in 4.3% (12/279) and multiple infections were 2.9% (8/279).

A total of 136 respiratory viral pathogens were identified from 105 patients. RSV was the most frequently detected (26.5%, 36/136), followed by PIV 22.1% (30/136), HRV 21.3 (29/136), CoV10.3% (14/136) and HAdV 9.6% (13/136). FluA, hMPV and BoCA were identified only 4.4%, 3.7%, and 2.2%, respectively. FluB and sH1N1 were not detected in this study. (Table 2).

**Table 2:** Viral etiologies identified.

Virus detected		Single infection(%)	Co- infection(%)	Multiple-infection (%)	Total (%)
		N = 85(30.5)	N = 12(4.3)	N = 8(2.9)	N = 105 (37.6)
**RSV**		28(10.0)	2(0.7)	6(2.2)	36(12.9)
	**A**	28(10.0)	1(0.4)	5(1.8)	34(12.2)
	**B**	0(0)	1 (0.4)	1(0.4)	2(0.7)
**PIV**		12(4.3)	10(3.6)	8(2.9)	30(10.8)
	**PIV1**	1(0.4)	2(0.7)	1(0.4)	4(1.4)
	**PIV2**	6(2.2)	2(0.7)	1(0.4)	9(3.2)
	**PIV3**	5(1.8)	6(2.2)	6(2.2)	17(6.1)
**HRV**		23(8.2)	4(1.4)	2(0.7)	29(10.4)
**CoV**		3(1.1)	3(1.1)	8(2.9)	14(5.0)
	**CoV OC43**	3(1.1)	0(0)	1(0.4)	4(1.4)
	**CoV NL63**	0(0)	2(0.7)	5(1.8)	7(2.5)
	**CoV HKU1**	0(0)	1(0.4)	1(0.4)	2(0.7)
	**CoV 229E**	0(0)	0(0)	1(0.4)	1(0.4)
**HAdV**		12(4.3)	1(0.4)	0(0)	13(4.7)
**Flu**		3(1.1)	1(0.4)	2(0.7)	6(2.2)
	**A**	3(1.1)	1(0.4)	2(0.7)	6(2.2)
	**B**	0(0)	0(0)	0(0)	0(0)
	**sH1N1**	0(0)	0(0)	0(0)	0(0)
**HMPV**		3(1.1)	1(0.4)	1(0.4)	5(1.8)
**BoCA**		1(0.4)	2(0.7)	0(0)	3(1.1)

In the co-infections, PIV accounted for 41.7% (10/24) and PIV3 was the most frequently detected. PIV and CoV equally accounted for 29.6% (8/27) of the multiple-infections. PIV3 and CoV NL63 were the predominant type. The majority (12/13) of HAdV was detected as single infection.

### Viral pathogens distribution in different age group

Table 3 shows the distribution of viral etiologies according to different age. RSV, HRV, PIV and CoV were detected in all groups. RSV was the most common pathogen in the patients under 1 year. The rates of RSV(17/153) and HRV(17/153) were similar in patients with the age between 1∼6 years. 11 of the 13 HAdV cases and all of the patients with hMPV infected also belonged to this group.

**Table 3:** Viral pathogens distribution in different age group.

Virus detected	<1(N = 88)		1–6(N = 153)		6–12(N = 38)	
	n	%	n	%	n	%
**RSVA&B**	17	19.3	17	11.1	2	5.3
**PIV(1–3)**	7	8	15	9.8	8	21.1
**HRV**	8	9.1	17	11.1	4	10.5
**COV**	7	8	3	2	4	10.5
**HAdV**	0	0	11	7.2	2	5.3
**FluA&B**	1	1.1	5	3.3	0	0
**HMPV**	0	0	5	3.3	0	0
**BoCA**	1	1.1	2	1.3	0	0
**Single infection**	21	23.9	57	37.3	7	18.4
**Co- infection**	5	5.7	6	3.9	1	2.6
**Multiple-infection**	3	3.4	2	1.3	3	7.9
**Positive cases**	29	33	65	42.5	11	28.9

### Viral pathogens distribution in different season

**Figure 1:**
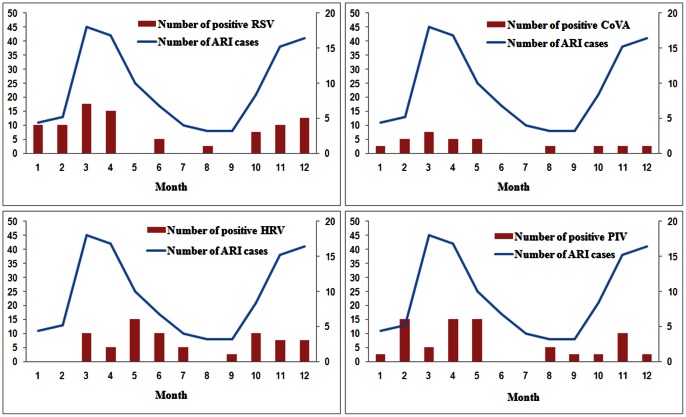
Viral pathogens distribution in different season.

The respiratory viruses circulated in 2011 in Gansu Province were presented in Figure 1. RSV showed seasonal variation with peaks during winter and spring. HRV was detected more frequently from May to June. Seasonality of PIV infection was apparent during February to May. CoVs were distributed throughout the year except in June, July and September and did not show obvious seasonality. Other viral pathogens appeared sporadically during the year.

## Discussion

This was the first study to detect multiple respiratory viruses in hospitalized children with ARIs in Lanzhou, Gansu Province, China. A total of 279 patients were enrolled from January to December 2011. Among the patients, 37.6% was positive for at least one virus, which was consistent with other studies (34.6%–62.6%) [3], [4], [5]–[16]. But it was a little lower than the studies performed in Beijing, China [17], which was probably due to the lower economic status, lower health awareness or environmental factors in Gansu Province.

Our findings were consistent with other reports from Asia and China: RSV was the dominant cause of respiratory tract infection in children under 5 years [18]–[23]. RSV A was much more common than RSV B (34/2). RSV was reported to be responsible for 33.8 million cases of lower ARI in 2005, of which 96% was in developing countries [24]. It would be important for local pediatricians to use antibiotics cautiously when children are hospitalized with ARIs.

HRV has been known to be responsible for upper ARIs as well as for some lower respiratory infection in children [25]–[28]. Our findings showed the presence of HRV in all age groups. Most HRVs (23 of 29) were commonly detected as single infection, which agreed with the report of Bezerra et al [29]. The rest of the cases were co-infections with PIV, RSV.

HAdV was another important virus detected as single infection; only one case was detected as co-infection with HRV. It differed from the conclusion of Bezerra et al [29] in which HAdV was the most common pathogen in co-infection. HAdV was reported to be responsible for 5–10% of ARI in children [11]. Our findings confirmed this report, showing a detection rate of 9.6 % (13/136), with 11 of the 13 patients infected by HAdV were under six years old.

The high detection rate of PIV and CoV in co-infections and multiple infections was an interesting finding. PIV was present in 50% (12/24) of co-infections. In particular, PIV3 and CoV NL63 were the most common types found. The much higher detection rate of CoV NL63 differed from previous epidemiological studies, in which CoV 229E and CoV OC43 were the most common types [30]. It is possible that the other types of CoV were not circulating in the area during the period of study. Meanwhile, the findings suggest that nosocomial infection cannot be ruled out.

Although hMPV had low prevalence in this study, only five hMPV infection cases were detected and were all aged between 1 to 6 years, which was in agreement with previous studies [31], [32].

We found that RSV was detected mainly during winter and spring, which corresponds with the cold and dry seasons. Typical continental climate with cold and dry winters, and the large difference between the indoor and outdoor temperatures in Gansu Province can easily induce respiratory diseases.

A multiple RT-PCR method was used in this study to detect sixteen common respiratory viruses [33]. This method was economical and fast in obtaining pathogen information, and both its sensitivity and specificity have been confirmed. However, there were some limitations in the current work: 1) a study spanning only one year and covering a single sentinel surveillance hospital cannot recruit enough samples to analyze one particular pathogen; 2) only viral pathogens were detected in our study; bacterial pathogens that cause ARIs were not included, which prevented us from getting comprehensive data on the pathogens that cause ARIs in this region.

In conclusion, this study provided background information concerning the respiratory viral etiology in Lanzhou area of Gansu Province. Our findings could serve as a reference for local CDC in drawing up further plans to prevent and control ARIs. They will also help clinicians to choose medicines for patients with ARIs. Moreover, the use of multiple RT-PCR makes rapid, effective, and affordable detection for virus a reality in resource limited areas.

## Materials and Methods

### Study population

Between January 1st and December 31st, 2011, patients who met the inclusion criteria of ARI (aged under 12 years) were enrolled from the First Clinical Medical College of Lanzhou University, a large-scale general hospital in Lanzhou City, China.

All patients were hospitalization and eligible for an onset of illness within 5 days.

### Definitions

1. Patients with ARIs must meet at least one of the inclusion criteria as follows:
  1) fever (axillary temperature≥37.2);
  2) cough, sore throat, wheeze, expectoration, chest pain;
  3) moist/dry rales;
  4) X-ray examination of lung inflammation showed punctate, patchy or uniform density shadow.
2. Single infection means only one viral pathogen detected in one sample; Co-infection means there are 2 viral pathogens detected in one sample; Multiple-infection means more than 3 viral pathogens detected in one sample.

### Specimen and data collection

A total of 279 throat swab specimens were taken from 279 inpatients. The throat swab specimens were collected by attending physician in 2-mL viral transport media, transported at 2°C–8°C and preserved at −80°C until to Institute for Viral Disease Control and Prevention of the Chinese Center for Disease Control and Prevention (CDC), Beijing.

Clinical information including demographic characteristics, symptoms, signs treatments, duration of hospitalization, clinical diagnosis were documented in case report forms (CRFs).

This study was approved by the Ethics Review Committee of the Chinese CDC and all participants gave a signed informed consent.

### Extraction of nucleic acids and multiplex PCR

The viral nucleic acid was directly extracted from the clinical specimens by using a QIAamp mini viral RNA extraction kit (Qiagen, Valencia, CA). RT-PCR was performed using a Qiagen OneStep RT-PCR kit (Qiagen, Valencia, CA) and GeneAmp 9700 thermal cycler (Applied Biosystems, Carlsbad, CA, USA). 25-μL reaction mixture contained 5 μL 5× PCR Mix, 1 μL of dNTP Mix, 1 μL of Enzyme Mix,1.25 μL of each primer mix(listed in Table 4), 0.1 μL of RNase inhibitor, 10.9 μL of RNase-DNase free water and 2 μL of template RNA. PCR reaction conditions included an reverse transcription at 50°C for 30 min, initial denaturation at 95°C for 15 min, followed by the first 10 cycles of denaturation at 95°C for 30 s, annealing at 55°C for 30 s, extension at 72°C for 30 s; the second 10 cycles of denaturation at 95°C for 30 s, annealing at 65°C for 30 s, extension at 72°C for 30 s; the third 30 cycles of denaturation at 95°C for 30 s, annealing at 48°C for 30 s, extension at 72°C for 30 s; and a final extension at 72°C for 7 min. [33]. The PCR products were analyzed by capillary gel electrophoresis (QIAxel DNA High Resolution kit) and the reference size were shown in Table 4. The detection of 16 respiratory viral pathogens was done in two groups: Group A was tested for the presence of FluA, FluB, sH1N1, HRV, CoV 229E/CoV OC43/CoV HKU1, PIV1, and HAdV; Group B was tested for the presence of RSVA/B, CoV NL63, PIV2/PIV3, HMPV and BoCA.

**Table 4:** Primers information in this study.

Target virus	Gene	Final concentration	Amplification size(bp)
HRV	5′-UTR	1 uM	144
FLuB	M gene	1 uM	166
CoV 229E	N gene	1 uM	182
CoV OC43	N gene	1 uM	200
CoV HKU1	N gene	1 uM	219
sH1N1	HA gene	1 uM	250
FluA	M gene	1 uM	270
PIV1	HA gene	1.75 uM	283
ADV	Hexon gene	1.75 uM	338
RSVA	F gene	1 uM	158
CoV NL63	Polymerase	1 uM	174
PIV2	HA gene	1 uM	193
HMPV	L gene	2 uM	207
PIV3	HA gene	1.5 uM	229
RSVB	F gene	2 uM	278
BOCA	NP1	2 uM	290

### Data analysis

The count data adopted constituted the ratio and frequency table description, and a non-normal distribution of measurement data using the median to describe the central tendency and percentiles to describe the discrete tendency. The group ratios were compared using Pearson's 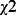 test, with an inspection level α of 0.05.
