# Correction: Viral Etiology of Acute Respiratory Infection in Gansu Province, China, 2011

**DOI:** 10.1371/annotation/91d141f8-549c-475c-891a-5d8b4e5f91fd

**Published:** 2013-07-30

**Authors:** Guohong Huang, Deshan Yu, Naiying Mao, Zhen Zhu, Hui Zhang, Zhongyi Jiang, Hongyu Li, Yan Zhang, Jing Shi, Shuang Zhang, Xinhua Wang, Wenbo Xu

The authors would like to provide some additional relevant information about this study that was not included in the published article:

The author Wenbo Xu is an inventor for a patent on the methodology employed for the detection of the viruses tested in the study (application number: 201110168721.X).

This methodology employed for the analyses followed the protocol described in the *BMC Infectious Diseases* article below:

*BMC Infect Dis*. 2012 Aug 14;12:189. doi: 10.1186/1471-2334-12-189.

The development of a GeXP-based multiplex reverse transcription-PCR assay for simultaneous detection of sixteen human respiratory virus types/subtypes.

Li J, Mao NY, Zhang C, Yang MJ, Wang M, Xu WB, Ma XJ.

This reference should have been cited in the article and the authors apologize for this omission.

The authors originally developed a method for the detection of viruses as described in the *Bing Du Xue Bao* article cited as reference 33. The original method used the GeXP strategy (chimeric primers) to detect 12 respiratory viruses. The protocol reported in the *BMC Infectious Diseases* article constitutes an improvement over the approach described in the *Bing Du Xue Bao* article as it combined the GeXP and Temperature Switch PCR strategies to detect 16 respiratory viruses.

The Temperature Switch PCR method had been originally described in the publication below, which should also have been cited in the article:

*BMC Genomics*. 2009 Dec 3;10:580. doi: 10.1186/1471-2164-10-580.

Temperature switch PCR (TSP): Robust assay design for reliable amplification and genotyping of SNPs.

Tabone T, Mather DE, Hayden MJ.

